# Common Disease, Multiple Rare (and Distant) Variants

**DOI:** 10.1371/journal.pbio.1000293

**Published:** 2010-01-26

**Authors:** Richard Robinson

**Affiliations:** Freelance Science Writer, Sherborn, Massachusetts, United States of America

**Figure pbio-1000293-g001:**
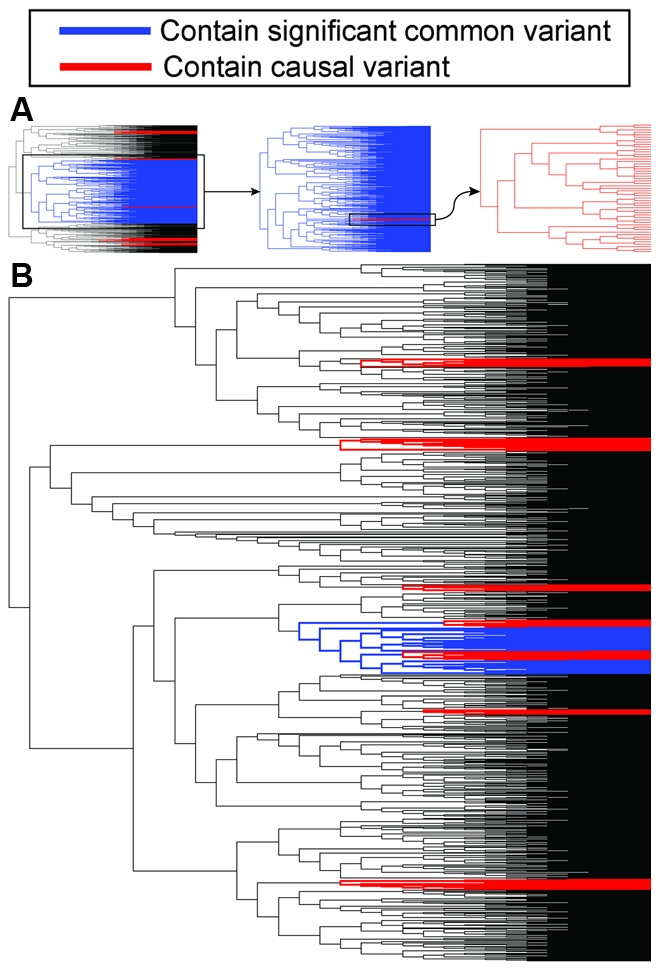
Genome-wide association studies have implicated hundreds of common variants of miniscule effect in diverse traits but a new study suggests that rare variants of much stronger effect may underlie some of these associations. Thus, large-scale sequencing may uncover more definitive leads about disease pathophysiology than the study of common variation has afforded.

[Fig pbio-1000293-g001]Genome-wide association (GWA) studies have emerged as a potentially powerful tool for discovery of new genes for common diseases, such as Alzheimer's disease and stroke. But the common interpretation of GWA findings might be incorrect in many cases, according to a new study by Samuel Dickson, David Goldstein, and colleagues in this issue of *PLoS Biology*. Their results suggest that the signals in these studies may not always be pointing to a few common gene variants, as assumed by most researchers, but instead to many rare variants, each of which causes relatively few cases, and each of which may be relatively far away from the site identified in the GWA study.

A GWA study compares DNA sequence variations across the genome in people with a particular disease to those without it. The “gene chips” on which the study is conducted typically analyze half a million or more “single nucleotide polymorphisms,” or SNPs (pronounced “snips”), looking for sequence differences between those with and those without the disease. The results of a GWA study are a handful of SNPs “associated with” the disease. The sequence change in a SNP itself is rarely if ever the actual cause of the disease, rather it acts as a signpost, a signal that there is some causative gene variation nearby. Therefore, the study results are the beginning, not the end, of the search for the causative gene or genes.

To date, GWA studies have identified hundreds of signposts associated with disease. But the search for causative genes derived from them has been remarkably less successful, with only a handful of causative genes discovered in follow-up sequencing studies. As well, many of the genes found to date have had only a weak effect—increasing the risk of disease, but only slightly, and therefore explaining only a small proportion of the disease.

It has been customary to assume that the variants being sought are common to many individuals with the disease (the so-called “common disease, common variant” hypothesis), and the difficulty in finding culprit genes was that these modest effects make the genes very difficult to recognize.

But an alternative explanation is also possible, that the disease is caused by multiple strong-effect variants, each of which is found in only a few people (the “common disease, many rare variants” hypothesis). Instead of the common signpost pointing to a common weak-effect variant, it might be pointing to many strong-effect variants. To distinguish this scenario from the common interpretation, the authors refer to associations between rare higher-impact variants and common markers as “synthetic associations”.

In the world of synthetic associations, the causal variant or variants may escape detection not because lots of people have the same weak-effect variant, but because a few people have one strong-effect variant, a few have another, and so on. Subsequent sequencing to discover the cause of the disease would be stymied because sequencing is usually done in only a few individuals, who may possess different causative variants. You wouldn't find a single common variant, and the multiple rare variants would look like noise.

To test the likelihood of synthetic associations, the authors conducted a simulation of 10,000 genotypes sprinkled with multiple rare variants for a disease, spread at some distance from a particular SNP, and assessed the possibility that the SNP would display an association with the disease. They found that an association was not only possible, but likely—about one third of the simulations detected a synthetic association to the SNP indistinguishable from the signal typically seen in real GWA studies. The strength of the signal increased as the number of individual causative variations increased and as the disease-causing potential of each increased. The association remained strong even when the model DNA regions were allowed to undergo recombination between chromosomes, potentially separating the variant from the SNP.

Of most significance for interpreting real GWA studies was that the causative variants could be located at a relatively large distance from the SNP—up to 2 megabases—and still contribute to the association. This is several times further away from the SNP marker than has usually been assumed, and encompasses a region containing scores of genes. When they examined a real dataset for rare mutations causing hearing loss, they found the same potential for synthetic associations to SNPs quite distant from the causative mutations.

The consequence, the authors suggest, is that sequencing near the SNP to find “the” causative gene will often be fruitless, and many causative genes will be missed if that is the only approach taken.

The alternative, whole-genome sequencing, is becoming increasingly practical, and offers the possibility of finding any variant, no matter how far away. But how will it be possible to pick out the needle of a causative variant in the haystack of genomic variability, if it is no longer right next to the signpost? Under the assumption that the variant exerts only a weak effect, it probably wouldn't be. Weak effects are thought to be due to subtle changes that still retain functionality of the encoded protein, like a dimmer switch on a light bulb. The genome is loaded with these kinds of variants, and most of them won't be involved in the disease.

But the weak-effect assumption may be wrong as well, since it rests on the assumption that the variant is common. If instead the variant is rare, its effect could be strong—not just contributing to the disease, but causing it—more like an on-off switch, but one that only a few people have. In that case, the sought-after variant is likely to be a classic kind of mutation—a nonsense sequence, for example—that is easy to find.

If this model is correct, it suggests that a SNP association in a GWA study may be pointing not at one gene, but lots of them; that these genes are likely to have stronger and perhaps easier-to-understand effects than presumed; and that finding these genes is likely to be simpler than has been the case so far. If the authors are right that some of the signals are synthetic, GWA results may be of particular value in interpreting the results of whole-genome sequencing studies. Focussing attention on regions of the genome that show GWA signals may help to identify likely the causal variants amongst the millions of variants identified in any sequencing study.

**Dickson SP, Wang K, Krantz I, Hakonarson K, Goldstein DB (2010) Rare Variants Create Synthetic Genome-Wide Associations. doi:10.1371/journal.pbio.1000294**

